# Engagement With Web-Based Fitness Videos on YouTube and Instagram During the COVID-19 Pandemic: Longitudinal Study

**DOI:** 10.2196/25055

**Published:** 2022-03-08

**Authors:** Wuyou Sui, Jonathan Rush, Ryan E Rhodes

**Affiliations:** 1 Exercise and Health Psychology Lab Western University London, ON Canada; 2 Department of Human Development and Family Studies College of Health and Human Development Pennsylvania State University University Park, PA United States; 3 Behavioural Medicine Laboratory School of Exercise Science, Physical and Health Education University of Victoria Victoria, BC Canada

**Keywords:** eHealth, physical activity, adults, adherence, COVID-19, fitness, video, YouTube, Instagram, social media, longitudinal

## Abstract

**Background:**

The COVID-19 pandemic has drastically changed the physical activity (PA) landscape through the closures of gymnasiums, schools, and many outdoor spaces. Physical distancing guidelines have also reduced opportunity for PA. The popularity of free web-based home fitness videos on video hosting platforms (eg, YouTube and Instagram) has spiked during the pandemic. Many web-based fitness videos offer a convenient, accessible, and cost-effective means of engaging in PA through regularly posted videos or discrete programs. Notably, traditional PA programs often suffer from poor adherence and high dropout rates, despite many advantages over web-based workout programs (eg, equipment, feedback, and in-person engagement). Thus, notwithstanding clear advantages of these web-based fitness videos, their ability to maintain long-term engagement and adherence is unknown.

**Objective:**

We explored patterns of engagement (ie, views, likes, and comments) for channels posting daily or program-based web-based fitness videos since the declaration of COVID-19 as a pandemic, over 4 months. Our secondary objective was to examine potential moderators of engagement metrics.

**Methods:**

An environmental scan was used to identify eligible channels. Eligible channels were (1) freely available on YouTube or Instagram and (2) posted daily or weekday series workouts or offered quarantine-specific workout programs. Searches for eligible channels were conducted on June 1 and 4, 2020. Engagement metrics of views, likes, and comments were then collected from channels’ videos posted between March 11 and June 26 or 30, 2020, inclusive, on June 26 or July 8, 2020. A series of multilevel modeling analyses were conducted to examine longitudinal changes in each of the 3 outcome variables.

**Results:**

Ten channels were deemed eligible and included in analyses; 6 posted regularly, while the other 4 posted discrete workout programs. Multilevel models revealed that both views and likes significantly decreased across days. Visually, channels display the sharpest drop in engagement within the first week. Linear change estimate indicates that the number of views initially declined by 24,700 per day (95% CI –44,400 to –11,300, *P*=.01) on average across all the channels. Channels with more subscribers declined in their views, likes, and comments at a significantly higher rate than those with fewer subscribers (*P*≤.04). The day of the week a video is posted, “virality,” and content of a video appear to influence engagement. Integrating behavior change techniques and posting new and varied videos often may help garner further engagement with these videos. Future research should examine common elements of videos, which drive engagement.

**Conclusions:**

Despite raw engagement metrics, each channel demonstrated peak engagement with the initial video followed by decreased engagement with subsequent videos. As many countries maintain restrictions on traditional PA facilities owing to the COVID-19 pandemic, determining methods to improve engagement and adherence with web-based fitness videos becomes increasingly important.

## Introduction

The COVID-19 pandemic has scaled up to over 38 million cases and over 1 million deaths worldwide as of this writing [1]. When COVID-19 was declared a pandemic on March 11, 2020 [2], this led to considerable changes in daily life including social distancing, elimination of community gatherings, restricted travel, and general instruction to “stay home” [3]. The consequences have affected how people engage in recreation, as many parks and recreation facilities were closed [4,5]. Commercial gyms, group exercise classes, and sporting activities ceased to operate over safety concerns and many have failed to reopen owing to bankruptcy or continued restrictions [6,7].

As a result of these drastic shifts in opportunity for physical activity (PA), the population has altered daily PA behaviors [8]. Fitbit’s blog presentation of its users noted a 5% to 20% reduction in total steps across the globe during the early stages of the pandemic [9]. Early research has replicated this general finding in China [10], the United States [11,12], the United Kingdom [13], Canada [14], and Europe [15]. Furthermore, higher-intensity PA, such as regular exercise, appears to be particularly compromised [12,14,15]. For example, a recent survey of 3052 US adults found a clinically meaningful drop in PA among active participants (pre–COVID-19) who were socially isolating [12]. These findings are concerning because regular moderate to vigorous intensity PA was already low in prevalence among high-income countries before the COVID-19 pandemic [16], despite how essential this behavior is for mental and physical health [17]. An increased burden that this wave of inactivity will cause leaves the population and the health care system weakened and at higher risk as COVID-19 continues to plague many countries [18].

Despite general trends in physical inactivity, some people have reported increasing their PA or remaining stable [14,15]. For example, work by Rhodes et al [14] found that minutes spent in PA during the pandemic were moderately positively associated with personality traits (eg, activity-extraversion, habit, and identity) and weakly positively associated with demographic factors (eg, income, education, and dog ownership). Notably, one of the key predictors of this stability has been opportunities to engage in home exercise [14,19]. Early research examining home exercise delivered through videos or videoconferencing software has demonstrated effectiveness in both improving levels of PA [20,21] and in increasing motivation to be physically active [22]. Further, affordances for PA (equipment and media) in the home environment have always been a reliable correlate of behavior [23]; yet, the COVID-19 pandemic restrictions on recreation facilities and gymnasiums have likely resulted in a dramatic shift in its importance. In concert with this demand, some commercial fitness businesses have transitioned to web-based fitness programs as an alternative to traditional exercise options [24]. Many web-based workout providers are offering quarantine specific workouts in an attempt to serve a new group of viewers, or prior patrons who are looking for a way to maintain their activity. Web-based fitness programs, on platforms such as YouTube and Instagram channels offer many advantages over traditional fitness options such as cost-effectiveness (free with an internet connection and no or minimal equipment), convenience (autonomy of when to watch and many different options), accessibility (vast catalogue of home fitness workouts and routines suited for different populations, skill levels, modes of exercise, etc), and community (comments, likes or dislikes, and live streaming allow for a level of interactivity that can mimic the social aspects of other traditional workout programs). In fact, YouTube reported a quadrupling of daily views for exercise videos with “no equipment” or “home” in the title since declaration of the pandemic [25].

There are clear advantages of these web-based exercise programs in these unprecedented times; yet, there remain several unanswered questions about long-term effectiveness and engagement. These programs cannot emulate several elements of traditional exercise options, such as movements that require equipment (resistance training machines, bikes, rowing, etc), true in-person feedback, and individualization of training. It may be that dropout from these programs is high. Indeed, fast dropout of approximately 50% from traditional gymnasiums and recreation centers is a well-established phenomenon [26]. More information on the patterns and potential moderators of engagement of these pandemic home exercise workouts will assist in tailoring future workouts toward the more successful approaches (eg, challenges vs daily workouts and population-focused approaches) as well as a general understanding of long-term engagement.

Thus, the purpose of this study was to explore the pattern of engagement levels of 10 web-based freely available exercise channels that posted either daily or program-based fitness videos since the beginning of the COVID-19 pandemic. We explored total engagement and changes in engagement through views, comments, and likes across a period of 4 months during the pandemic. We further explored potential moderators of engagement metrics, such as viewership across a week and engagement across specific video posting formats and video content. Given the exploratory nature of this study, no formal confirmatory hypothesis was proposed. However, based on prior exercise adherence research, we expected to see that engagement with these web-based videos—via higher engagement metrics—would occur for the initial videos posted by channels; however, a sharp drop-off in engagement was also hypothesized to follow in subsequent videos, paralleling retention and adherence patterns in existing PA efforts [26].

## Methods

### Eligibility Criteria

Eligible media met the following criteria: (1) freely available on YouTube or Instagram and (2) posting daily or weekday series workouts or offering quarantine-specific workout programs, as these criteria were rationalized to parallel closest with regular gymnasium-going or exercise or PA guidelines [27]. As such, websites that offered free workout series or programs but were not hosted on YouTube or Instagram (eg, CrossFit [28]) were excluded, as comparable metrics of engagement (eg, views, likes, and comments) were not consistently available. Similarly, series or programs that were accessed through a proprietary app (eg, Gymshark conditioning workout app [29]) were also not included. Further, channels that posted regular videos but were not as part of a regular series or program (eg, vlogs, testimonials, and diet advice) were not included, as these unstructured or unrelated videos were thought to receive variable hits depending on their individual popularity (eg, trending or viral videos).

### Search Strategy

The following keywords were used (in combination) within search engines: “Daily,” “Online,” “Workout,” “Exercise,” “Program,” “Streaming,” “Fitness,” “At-Home,” “Of the day,” “Today’s date” (ie, date of the search), “Class,” “Session,” “quarantine,” “lockdown,” and “COVID-19.” For example, “Daily online workout program” was a permutation of search strategy used.

### Information Sources

Search term keywords and combinations therein were searched directly into a Google search engine, as well as YouTube and Instagram platform search engines. For Google searches specifically, webpages that contained lists of available web-based workouts (eg, “The best places to go for free online workouts” [30]) were parsed for eligible channels.

### Procedure

An environmental scan was initially employed to determine the media sources (ie, YouTube and Instagram channels) for longitudinal data extraction. According to Graham [31], environmental scans leverage diverse sources and amounts of data, so decision-makers can be informed of “current social, economic, technological, and political contexts, and to identify any potential short- and long-term shifts.” Importantly, the flexibility and diversity of potential data sources within an environmental scan can reveal preliminary trends or current relationships that may warrant further, more robust investigation. As such, two initial searches to identify eligible channels were conducted: one on June 1 and one on June 4, 2020.

Upon selection of appropriate YouTube and Instagram channels, overall engagement metrics were collected for videos posted between March 11 and June 26 or 30, 2020, inclusive. Specifically, these overall engagement metrics were collected on June 26 or July 8, 2020. While this extraction method does infer that older videos have more time to accumulate engagement, the regular or programmed nature of the videos posted by the observed channels is likely still sensitive enough to reflect changes in engagement over time.

### Primary Outcome: Engagement Metrics

Engagement metrics from videos of eligible channels were collected; specifically, number of views, number of likes (if available), and number of comments. Further, to explicate trends in video engagement, channels that posted a regular series of workout videos (ie, regularly) were charted differently than channels that released a discrete workout program (ie, program). Further, number of channel subscribers was collected as a potential moderator of channel engagement.

### Statistical Analyses

A series of multilevel modeling analyses were conducted to examine longitudinal changes in the engagement metrics. Separate models were carried out for each of the three outcome variables (ie, views, likes, and comments) and were predicted by a linear and quadratic time variable, coded as days since COVID-19 was declared as a global pandemic (March 11, 2020) [2]. Number of channel subscribers (grand-mean centered) and video start date were included as between-channel covariates to statistically adjust for initial differences in channel exposure and time elapsed between the first video and the onset of the pandemic. Subscribers and start date were also included as moderators on both linear and quadratic slopes to determine if the rate of change differed depending on channel size or video onset. All models were estimated in Mplus (version 8.4) using the Bayes estimator to facilitate model convergence. Statistical significance was set at *P*<.05.

## Results

### Channel Descriptives

Overall, 10 channels were observed in this environmental scan: Fit Factory [32], One Workout A Day [33], The Body Coach [34], Orange Theory Fitness [35], Planet Fitness [36], Holly Dolke [37], Six-Pack Factory [38], Leansquad [39], Amanda Bisk [40], and Gaby Pimental [41]. The majority of these channels were identified through websites found through Google searches that posted curated lists of web-based exercise videos or channels. Descriptive characteristics and statistics for each channel are presented in Tables 1 and 2, respectively. Of the 10 channels observed, 9/10 (90%) were based on YouTube and 1/10 (10%) was based on Instagram. The channels were almost evenly split between regularly posting videos (6/10, 60%) and posting discrete programs (4/10, 40%). Of the channels that posted regularly, post frequency ranged from daily videos (4/6, 67%) to weekdays only (2/6, 33%). Of the channels that posted programs, program length ranged from 7 days (2/4, 50%) to 30 days (1/4, 25%). With respect to posting date, the majority of channels observed began posting in March 2020 (8/10, 80%), with 1 (10%) channel starting in mid-May [32] and 1 (10%) channel starting in June [36]. Subscriber count varied drastically among channels, ranging from 766 [41] to 2,500,000 [34], with a median value of 74,100 (based on the date of data collection).

**Table 1:** Descriptive characteristics for each channel.

Channel	Channel characteristics				Subscribers, N		Videos posted, N		Video start date
	Platform	Posting format	Target audience						
Fit Factory	Instagram	Regularly (daily)	Adults	30,100		38		May 15, 2020	
One Workout a Day	YouTube	Regularly (daily)	Women	77,100		100		March 23, 2020	
The Body Coach	YouTube	Regularly (weekdays)	Children and parents	2,500,000		100		March 23, 2020	
Orange Theory Fitness	YouTube	Regularly (daily)	Adults	89,300		105		March 18, 2020	
Planet Fitness	YouTube	Regularly (weekdays)	Adults and adolescents	71,100		12		June 1, 2020	
Holly Dolke	YouTube	Program (30 days)	Women	1,110,000		30		March 23, 2020	
Six-Pack Factory	YouTube	Program (7 days)	Adults (men)	1,460,000		7		March 30, 2020	
Leansquad	YouTube	Program (7 days)	Adults	21,000		14		March 21, 2020	
Amanda Bisk	YouTube	Program (14 days)	Women	24,800		14		March 20, 2020	
Gaby Pimental	YouTube	Regularly (daily)	Older adults	766		30		March 18, 2020	

**Table 2:** Descriptive statistics for average video engagement metrics of each channel.

Channel^a^	Views, mean (SD)	Likes, mean (SD)	Comments, mean (SD)
Fit Factory	3396.3 (1679.4)	159.8 (67.4)	17.4 (10.8)
One Workout a Day	4164.4 (8787.1)	209.6 (327.5)	17.0 (15.8)
The Body Coach	1,110,476.1 (1,077,577.6)	23,462.3 (16,401.5)	853.7 (589.8)
Orange Theory Fitness	60,951.4 (37,330.2)	243.8 (244.5)	22.8 (15.9)
Planet Fitness	2652.0 (829.9)	53.3 (29.1)	0.00 (0.00)
Holly Dolke	140,494.2 (120,562.1)	5278.4 (4913.1)	512.0 (421.1)
Six-Pack Factory	9707.3 (7060.8)	299.9 (231.6)	123.7 (63.1)
Leansquad	6811.1 (8272.2)	89.7 (102.9)	8.9 (9.6)
Amanda Bisk	19,175.0 (21,117.2)	266.4 (328.9)	17.6 (10.7)
Gaby Pimental	1071.0 (1337.8)	15.6 (13.3)	6.7 (4.7)

^a^Grand means: 135,889.9 views, 3007.9 likes, and 158 comments.

### Engagement Metrics Across the COVID-19 Pandemic

**Figure 1:**
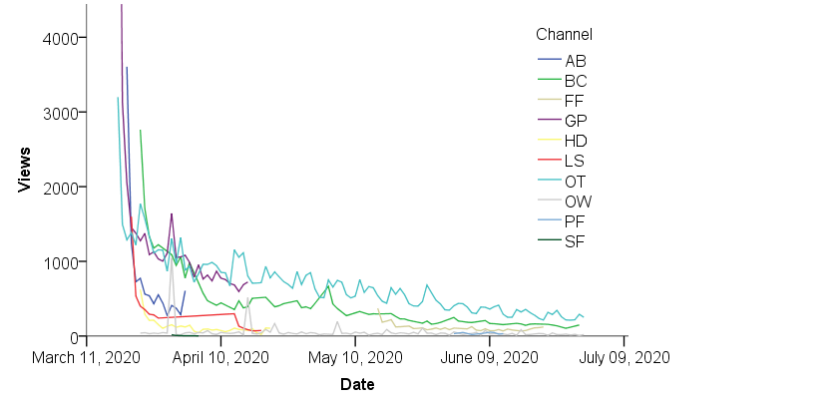
Trajectories of views for each channel during the COVID-19 pandemic. Values are presented per 1000 subscribers. AB: Amanda Bisk, BC: The Body Coach, FF: Fit Factory, GP: Gaby Pimental, HD: Holly Dolke, LS: Leansquad, OT: Orange Theory Fitness, OW: One Workout a Day, PF: Planet Fitness, SF: Six-Pack Factory.

The raw daily engagement metrics are displayed in Figure 1, Multimedia Appendix 1, and Multimedia Appendix 2. Multilevel models were used to formally test for the decline in engagement metrics throughout the pandemic. Our results revealed that both views and likes significantly decreased across days (see linear change estimate in Table 3 and Multimedia Appendix 3, respectively). These linear declines in views and likes were still detectable after adjusting for number of channel subscribers and video start date. Table 3, Multimedia Appendix 3, and Multimedia Appendix 4 also indicate that channels with more subscribers had significantly more views, likes, and comments on average than channels with fewer subscribers. Conversely, channels that began their videos later into the pandemic (ie, had a higher value for their video start date) produced fewer views and likes on average compared to channels that began their videos closer to the onset of the pandemic. Daily views were divided by 10,000 to enable model estimation. Therefore, the intercept value of 82.24 indicates that at the onset of the pandemic (time 0), the estimated number of views would be 822,400. The linear change estimate of –2.47 indicates that the number of views were initially declining by 24,700 per day, on average across all the channels. The positive quadratic change value indicates a slowing of this rapid decline in views. However, the quadratic term was not statistically significantly for any of the engagement metrics. Daily likes and comments were also scaled to enable model estimation (divided by 100 and 10, respectively). Therefore, the initial estimated number of likes would be 12,787 with an initial linear decline of 289 likes per day. Descriptively, visual inspection of Figure 1, Multimedia Appendix 1, and Multimedia Appendix 2 indicate the rapid decline in views, likes, and comments for most of the channels across time. Visual inspection also revealed potential “micro” patterns of engagement. For example, the Orange Theory Fitness channel appears to demonstrate a repeating weekly pattern of engagement, whereby engagement is highest on Monday and dips midweek before rising again during Friday and the weekend. Additionally, uncharacteristic spikes in engagement may indicate the “virality” of some videos.

**Table 3:** Daily changes in views during the COVID-19 pandemic.

Variable			Views^a^		
			Estimate (SE)	95% CI	*P* value
**Fixed effects**					
	Intercept		82.24 (36.13)	2.92 to 145.73	*.04^b^*
	Linear change		−2.47 (0.86)	−4.44 to −1.13	*.010*
	Quadratic change		0.02 (0.03)	−0.01 to 0.07	.09
	Subscribers^c^		0.14 (0.03)	0.07 to 0.21	*.004*
	Video start day^d^		−4.35 (3.53)	−12.35 to 1.08	.14
	Linear×Subscribers		−0.004 (0.00)	−0.005 to −0.002	*.004*
	Linear×Start day		0.11 (0.09)	0.01 to 0.32	*.03*
	Quad×Subscribers		0.00 (0.00)	−0.00 to 0.00	.06
	Quad×Start day		−0.00 (0.00)	−0.003 to 0.001	.16
**Random effects**					
	Within-person		605.88	525.54 to 692.55	N/A^e^
	**Between-person**				
		Intercept	4224.11	1075.70 to 2233.00	N/A
		Linear	62.71	0.04 to 1156.84	N/A
		Quadratic	0.05	0.01 to 1.30	N/A

^a^Views were divided by 10,000 to enable model estimation.

^b^Italicized values are significant at *P*<.05.

^c^Subscribers = number of channel subscribers / 1000.

^d^Start day = number of days from the beginning of the declaration of COVID-19 as a pandemic (March 11, 2020).

^e^N/A: not applicable.

### Moderators of Channel Engagement

**Figure 2:**
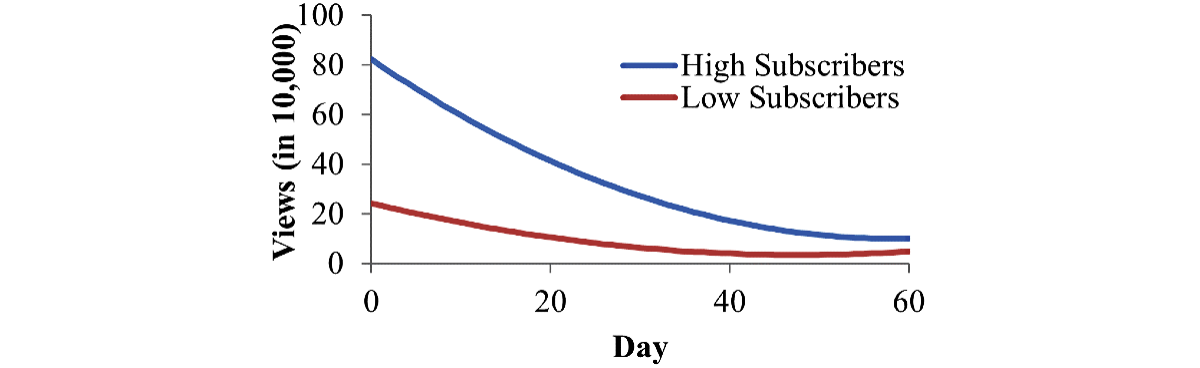
Estimated trajectories of views for channels with high (500,000) and low (100,000) subscribers.

The interaction between the number of channel subscribers and linear declines was significant for each of the 3 engagement metrics; that is, channels that had more subscribers experienced a decline in their views, likes, and comments at a significantly faster than those with fewer subscribers. Figure 2, Multimedia Appendix 5, and Multimedia Appendix 6 displays the nature of this interaction for a channel with 500,000 subscribers compared to that with 100,000 subscribers for views, likes, and comments, respectively. Inspection of the figures depicts how channels with more subscribers begin with more engagement but decline at a faster rate than those with fewer subscribers.

## Discussion

### Principal Findings

Our study sought to explore the pattern of engagement levels of web-based freely available exercise channels that posted regular or program-based videos since the beginning of the COVID-19 pandemic. To this end, our environmental scan identified 10 channels that met inclusion criteria. While the study is the first of its kind and thus exploratory, we expected to see a large engagement presence for videos posted closer to the start of the pandemic, followed by a sharp drop-off in engagement in subsequent videos, which mirrored patterns among traditional PA opportunities [26]. Overall, commensurate with our expectations, all the channels observed saw a significant linear decline for posted videos over time in views and likes, with a trending decline for comments. Visual inspection indicated the sharpest decline in these engagement metrics within the first week of when the channel started posting videos, controlling for start date, paralleling previously reported dropout rates among traditional gym use [26].

Specifically, earlier videos posted by a channel garnered more views, likes, or comments than subsequent videos, with a drop-off in engagement in subsequent videos, regardless of channel or posting format. The multilevel models also revealed the role of the video start date (in relation to the declaration of COVID-19 as a pandemic), in that channels that began posting videos closer to March 11, 2020, saw higher engagement than those that began posting later. These findings may be representative of the trend in popularity of web-based fitness videos throughout the course of the COVID-19 pandemic. Lockdowns and stay-at-home messaging in early March drastically increased the number of individuals who were staying at home [42], which coincided with a spike in interest in Google searches for “online fitness videos” and “online exercise videos” [43] and a quadrupling of views for “no equipment” and “home” exercise videos on YouTube [25]. However, return to pre–COVID-19 levels of interest in these search terms by May 2020 also generally coincides with plateaus in engagement metrics among videos posted by the observed fitness channels. It is possible that colder or inclement weather may have contributed to the greater engagement in these videos [44] closer to March and a diminishing of engagement over the next few months (ie, winter or spring transition in the Northern hemisphere), in that indoor opportunities for PA—such as web-based workout videos—may have been preferentially chosen over outdoor opportunities in the early months of the COVID-19 pandemic. However, the dramatic drop-off we observed is notably incongruent from the slower moderation of season on PA [44]. Hence, the overall trend we observed supports the notion that the COVID-19 pandemic has driven a mass of engagement to web-based fitness and exercise videos on YouTube and Instagram.

Notably, while our multilevel models revealed a significant negative linear relationship between the video posting date and engagement metrics, when examining our data descriptively, the sharpest decline in all engagement metrics was evident within a week of a channel posting its first video, regardless of the start date. This initial drop-off in channel engagement parallels the poor adherence rates to traditional PA programs, whereby 50% of adults drop out within a few months of beginning a PA program [26]. However, use of YouTube and Instagram as the sole means of delivering a PA program presents several disadvantages to a traditional PA program, which may explain the sharper decrease in engagement. By virtue of the medium of delivery (ie, posting a video on the internet), the web-based fitness videos examined do not incorporate many of the strategies that have been shown to improve adherence to PA. For example, viewers of web-based fitness videos do not inherently have a way to receive feedback on the basis of their participation, which can lead to overexertion or improper form or injury, both of which have been shown to deter future participation in PA [45]. Further, capacity for elements such as goal-setting and social participation with other viewers is limited with web-based fitness videos given the asynchronous “delivery” of the workout.

Descriptively, several potential moderators for engagement were identified within the data. Specifically, while the larger trend of engagement demonstrated was a negative linear relationship, smaller patterns within the data were also observed through visual inspection. For example, some “regularly” posting channels appeared to demonstrate weekly patterns in video views; specifically, video views were higher at the beginning of the week (ie, Monday) and tended to decline throughout the week. This weekly—or circaseptan—cycle has been previously documented for smoking cessation efforts [46] and other health-related behaviors [47], whereby the healthiest contemplations occur during Monday. The Orange Theory Fitness channel [35], in particular, appeared to demonstrate an M-shaped circaseptan cycle, with relatively higher engagement at the start and end of the week. Other notable patterns include large spikes in engagement metrics, which are may be attributable to the “virality” of some videos, or videos that become popular through sharing and resharing [48]. These viral videos do not usually deviate from the creator’s typical content, but rather feature some characteristic or characteristics that facilitate traction on social media very soon after publication. Smaller spikes in engagement were also present in regularly posting channels and may be reflective of the specific content of the PA that day. In other words, the specific content of that day’s workout may elicit more engagement from viewers than typical or expected.

In general, patterns of engagement metrics were related to other engagement metrics. For example, views of a video were consistently several magnitudes higher than likes on a video; similarly, the number of likes was generally an order of magnitude higher than comments on a video. These findings are unsurprising given that these metrics likely represent an increasing level of engagement of a video, respectively. Views of a video represent a spectrum of viewers: from those who watch the full video (and presumably participate in the PA) to those who may only have a passing curiosity in the video and disengage after 30 seconds of watching the video (ie, minimum watch time to be considered a view [49]). On the other hand, likes and comments are intentionally performed by the viewer, which may be indicative of higher engagement with the video.

Of the channels observed, adults were the primary audience for these videos, followed by women, children and adolescents, and then older adults. Despite insufficient observations to statistically compare channels examining these groups, descriptively, channels demonstrate fairly consistent patterns of engagement. Perhaps a notable exception to this is the channel focused on older adults [41]; Gaby Pimental’s channel, despite a subscriber count of <1000, had some of the highest views, likes, and comments per subscriber. Whether this is owing to the low subscriber count (ie, less engagement is needed to achieve higher ratio of engagement) or potentially inherent differences to how older adults engage with these videos (eg, more likely to like and comment compared to other demographics) is worthy of future investigation.

### Practical Implications

This work holds several potential practical implications for both future digital exercise research and viewers or creators of digital exercise content. Our data suggest that COVID-19 has generated interest and engagement with web-based fitness and exercise videos. Furthermore, as reports of resurgences of COVID-19 cases emerge globally and many countries revert to lockdowns and stay-at-home orders—and consequently, limitations to traditional PA opportunities—interest in these web-based fitness and exercise videos is likely to rise again. Given the similar drop-off in adherence between the web-based exercise videos observed and traditional PA opportunities (eg, gymnasiums and recreation centers), research investigating means by which these videos can retain engagement with new viewers may be able to draw from existing exercise adherence research; for example, for content creators, posting videos often and regularly, encouraging habit formation [50], posting a variety of exercises [51], responding to viewers’ comments as a means of social support and feedback [52], and offering incentives or prizes for regular engagement [53] may all assist content creators in improving and retaining their subscriber base. Similarly, for viewers and subscribers to these channels, goal-setting and creating an action plan on the basis of the posting schedule of a channel may improve adherence [54], and more distally, habit formation [54]. Further, liking and commenting on videos and engaging with the content creator may improve engagement and adherence through fostering social identity [55]. Additionally, exploring avenues by which these videos can be made more accessible or available to digitally disadvantaged populations (eg, low-income households and those with poor digital literacy) is worthy of pursuit, as currently, engagement with these videos—and any benefits imparted from PA derived from these videos—is limited by digital inequities [56].

By extension, research investigating how engagement with these types of videos is linked to actual exercise behavior is also crucial for understanding their effectiveness. Further, building on previous work examining engagement with YouTube videos [57], content analyses examining common elements of more popular exercise videos can reveal potentially unique factors driving engagement in this medium of PA promotion, such as branding, targeting emotions or affective states, and authenticity [58,59]. Similarly, determining demographics for the audiences of these videos can reveal which type of channel or video may be more effective for promoting PA among different populations [58].

### Limitations

There are limitations to our work and its interpretation. The primary limitation is that we cannot ascertain the extent to which any individual engagement metric, or combination thereof, translates to actual PA. YouTube only requires 30 seconds of video watch time to be considered a view [49]; similarly, likes and comments—while necessitating more engagement from the viewer—can also be done without participating in any actual exercise. Notably, likes and comments can also only be left by viewers with a YouTube or Instagram account, which considerably limits the use of solely these metrics as a measure of video engagement. Perhaps the best surrogate measure for actual exercise participation may be average watch duration for a video, since it is unlikely for an individual to watch a 30-45–minute workout video without participating in exercise themselves. Unfortunately, these metrics are only available to the channel owner, along with other useful metrics, such as audience retention (ie, percentage of video watched), number of shares, demographics, and more specific engagement metric trends (eg, changes in day-to-day engagement). Further, some channels posted their videos “live” initially (eg, The Body Coach [34]), and hence have comments and other engagement metrics that are not archived on the webpage. Engagement metrics from these “live” sessions may also provide a better representation of exercise participation; however, these are also only available to the channel owner. Our work also only examined a narrow criterion of freely available channels on YouTube or Instagram. Our searches uncovered numerous more “popular” channels, based on subscriber count and views or uploads, which did not meet our eligibility criteria. Inclusion of these channels through broader inclusion criteria may reveal additional patterns of engagement not observed in this study. Comparably, other platforms (eg, Twitter, Facebook, and paid platforms) may include features to influence engagement beyond what we observed. However, whether metrics for engagement are as easily extracted from these platforms remains an area for future research. Finally, the mode of delivery for these videos (ie, YouTube and Instagram) presents as another limitation, as these videos are only available to individuals with access to these websites. As the COVID-19 pandemic continues to exacerbate digital inequalities [56] (eg, the ability to receive PA promotion and facilitation via web-based exercise videos) the generalizability of these findings is limited to those with access to said videos.

### Conclusions

In summary, the declaration of COVID-19 as a global pandemic has coincided with a surge in engagement (ie, views, likes, and comments) with web-based fitness and exercise videos on YouTube and Instagram, with observed videos posted closer to the start of the pandemic garnering significantly more engagement than subsequent videos. However, compared to earlier posted videos, engagement metrics associated with subsequent videos decline significantly, regardless of posting type, with the sharpest decline in engagement occurring within a week of the initial video and greater decline for channels with higher subscriber counts. Investigating means by which these types of channels can improve and maintain engagement with viewers, elements of current popular videos that garner engagement, as well as how this engagement translates to actual exercise behavior, are all lucrative areas for future research, especially throughout the uncertainty associated with the COVID-19 pandemic.

## Appendix

Multimedia Appendix 1 Trajectories of likes for each channel during the COVID-19 pandemic.

Multimedia Appendix 2 Trajectories of comments for each channel during the COVID-19 pandemic.

Multimedia Appendix 3 Daily changes in likes during the COVID-19 pandemic.

Multimedia Appendix 4 Daily changes in comments during the COVID-19 pandemic.

Multimedia Appendix 5 Estimated trajectories of likes for high (500,000) and low (100,000) subscribers.

Multimedia Appendix 6 Estimated trajectories of comments for high (500,000) and low (100,000) subscribers.

## Abbreviations

PA: physical activity
