# Supplementary material for: Engagement With Web-Based Fitness Videos on YouTube and Instagram During the COVID-19 Pandemic: Longitudinal Study
Source: JMIR Form Res. 2022 Mar 8;6(3):e25055. doi: 10.2196/25055 (PMC8906834; doi:10.2196/25055)

**Multimedia Appendix 2**. Trajectories of comments for each channel during the COVID-19 pandemic. Note. Values are presented per 1000 subscribers.


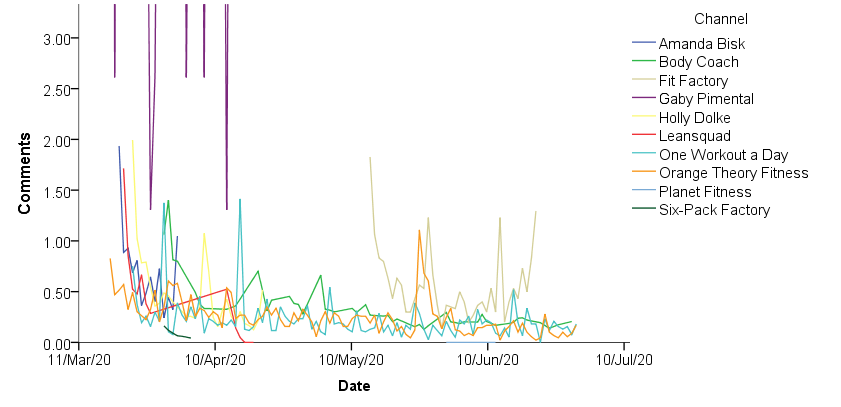

Supplement: Multimedia Appendix 2 [file formative_v6i3e25055_app2.docx]
