# Supplementary material for: Engagement With Web-Based Fitness Videos on YouTube and Instagram During the COVID-19 Pandemic: Longitudinal Study
Source: JMIR Form Res. 2022 Mar 8;6(3):e25055. doi: 10.2196/25055 (PMC8906834; doi:10.2196/25055)
Supplement: Multimedia Appendix 3 [file formative_v6i3e25055_app3.docx]

**Multimedia Appendix 3**. Daily changes in likes during the COVID-19 pandemic.

|  | Likes^a^ | | |
| --- | --- | --- | --- |
| Variable | Estimate (*SE*) | 95% CI | *P*-value |
| Fixed Effects |  |  |  |
| Intercept | 127.87 (42.62) | [28.15, 195.35] | *.02* |
| Linear change | −2.89 (1.99) | [−7.11, −0.02] | *.049* |
| Quadratic change | 0.01 (0.05) | [−0.03, 1.26] | .46 |
| Subscribers^b^ | 0.19 (0.04) | [0.10, 0.25] | *<.001* |
| Video start day^c^ | −8.25 (5.31) | [−19.86, −1.40] | *.010* |
| Linear*Subscribers | −0.003 (.001) | [−0.006, −0.001] | *.04* |
| Linear*Start day | 0.23 (0.14) | [0.04, 0.52] | *.02* |
| Quad*Subscribers | <0.01 (0.00) | [−0.00, 0.00] | .45 |
| Quad*Start day | −0.00 (0.00) | [−0.004, 0.001] | .10 |
|  |  |  |  |
| Random Effects |  |  |  |
| Within-person | 3622.83 | [3121.04, 4219.14] |  |
| Between-person |  |  |  |
| Intercept | 3414.35 | [412.36, 24516.48] |  |
| Linear | 37.30 | [0.02, 517.98] |  |
| Quadratic | 0.09 | [0.00, 9.05] |  |

^a^Likes were divided by 100 to enable model estimation.

^b^Subscribers = number of channel subscribers / 1000.

^c^Start day = number of days from the beginning of the declaration of COVID-19 as a pandemic (March 11, 2020).
