# Supplementary material for: Engagement With Web-Based Fitness Videos on YouTube and Instagram During the COVID-19 Pandemic: Longitudinal Study
Source: JMIR Form Res. 2022 Mar 8;6(3):e25055. doi: 10.2196/25055 (PMC8906834; doi:10.2196/25055)
Supplement: Multimedia Appendix 4 [file formative_v6i3e25055_app4.docx]

**Multimedia Appendix 4.** Daily changes in comments during the COVID-19 pandemic.

|  | Comments^a^ | | |
| --- | --- | --- | --- |
| Variable | Estimate (*SE*) | 95% CI | *P*-value |
| Fixed Effects |  |  |  |
| Intercept | 55.96 (20.99) | [12.04, 98.61] | *.02* |
| Linear change | −1.64 (1.19) | [−4.71, 0.15] | .07 |
| Quadratic change | 0.01 (.03) | [−0.02, 1.00] | .37 |
| Subscribers^b^ | 0.10 (0.02) | [0.05, 0.13] | *.02* |
| Video start day^c^ | −0.31 (1.94) | [−4.13, 3.33] | .87 |
| Linear*Subscribers | −0.003 (.001) | [−0.006, −0.001] | *<.001* |
| Linear*Start day | 0.002 (0.06) | [−0.12, 0.13] | .96 |
| Quad*Subscribers | 0.00 (0.00) | [−0.00, 0.00] | .30 |
| Quad*Start day | −0.00 (0.00) | [−0.002, 0.002] | .98 |
|  |  |  |  |
| Random Effects |  |  |  |
| Within-person | 275.22 | [236.81, 319.42] |  |
| Between-person |  |  |  |
| Intercept | 1276.70 | [15.01, 10268.08] |  |
| Linear | 41.79 | [1.83, 371.21] |  |
| Quadratic | 0.13 | [0.002, 2.94] |  |

^a^Comments were divided by 10 to enable model estimation.

^b^Subscribers = number of channel subscribers / 1000.

^c^Start day = number of days from the beginning of the declaration of COVID-19 as a pandemic (March 11, 2020).
