# Supplementary figures and images for: Engagement With Web-Based Fitness Videos on YouTube and Instagram During the COVID-19 Pandemic: Longitudinal Study
Source: JMIR Form Res. 2022 Mar 8;6(3):e25055. doi: 10.2196/25055 (PMC8906834; doi:10.2196/25055)

**Multimedia Appendix 5.** Estimated trajectories of likes for high (500,000) and low (100,000) subscribers.


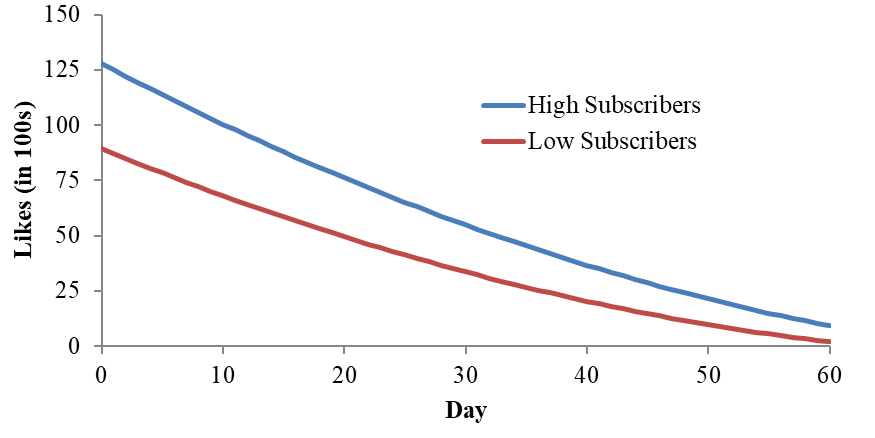

Supplement: Multimedia Appendix 5 [file formative_v6i3e25055_app5.docx]

**Multimedia Appendix 6**. Estimated trajectories of comments for high (500,000) and low (100,000) subscribers.


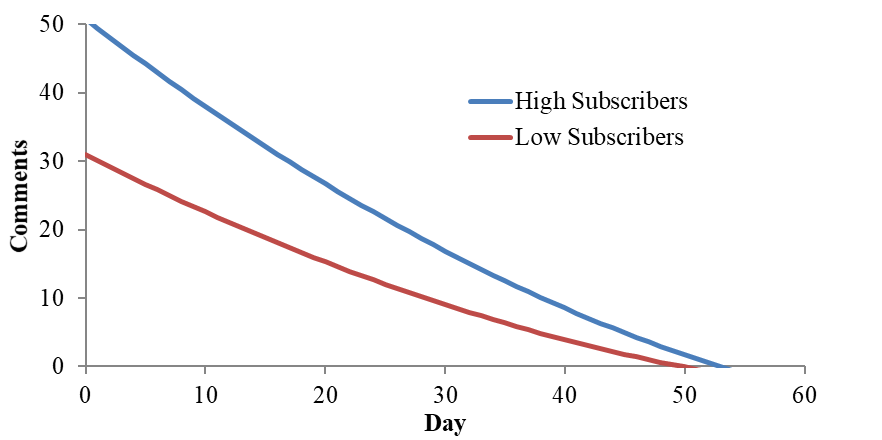

Supplement: Multimedia Appendix 6 [file formative_v6i3e25055_app6.docx]
